# Topical corticosteroid phobia in parents of pediatric patients with atopic dermatitis: a multicentre survey

**DOI:** 10.1186/s13052-017-0330-7

**Published:** 2017-02-28

**Authors:** Maya El Hachem, Francesco Gesualdo, Giampaolo Ricci, Andrea Diociaiuti, Loredana Giraldi, Orsola Ametrano, Corrado Occella, Anna Belloni Fortina, Mirella Milioto, Fabio Arcangeli, Oriana Simonetti, Simona Giancristoforo, Elisabetta Calamelli, Carlo Mazzatenta, Iria Neri

**Affiliations:** 1 Pediatric Dermatology Unit, Bambino Gesù Children’s Hospital, IRCCS, Rome, Italy; 2 Multifactorial Disease and Complex Phenotype Research Area, Bambino Gesù Children’s Hospital IRCCS, Rome, Italy; 3 Department of Medical and Surgical Sciences, Pediatric Unit, S. Orsola-Malpighi Hospital - University of Bologna, Bologna, Italy; 4 Pediatric Dermatology, AORN Santobono-Pausilipon, Naples, Italy; 5 Dermatology Unit, Istituto Giannina Gaslini, Genoa, Italy; 6 Department of Medicine, Pediatric Dermatology Unit, University of Padua, Padua, Italy; 7 Department of Medicine, Unit of Pediatric Dermatology, ARNAS Civico, Palermo, Italy; 8 Dermatology Unit, M. Bufalini Hospital, Cesena, Italy; 9 Department of Clinical and Molecular Sciences, Clinic of Dermatology, Università Politecnica delle Marche, Ancona, Italy; 10 Dermatology Unit, San Luca Hospital, ASL 2, Lucca, Italy; 11 Division of Dermatology, Department of Specialised, Experimental and Diagnostic Medicine, University of Bologna, S. Orsola-Malpighi Hospital, Bologna, Italy

**Keywords:** Atopic dermatitis, Topical corticosteroids, Children, Corticophobia

## Abstract

**Background:**

Families of children affected with atopic dermatitis (AD) often report fear and anxiety regarding treatment with topical corticosteroids (TCS), which may lead to reduced compliance. The objective of our study was to measure, through a standardized questionnaire, fear of TCS in families of pediatric patients with AD and to identify items associated with fear.

**Methods:**

Families of pediatric patients with AD were enrolled in 9 Italian centers of pediatric dermatology. Enrolled parents were invited to fill in a questionnaire including questions on sociodemographic and clinical characteristics and 3 sets of questions on corticosteroid phobia (general fear, specific fears, behaviours regarding TCS). Determinants of the level of general fear were investigated through multivariable analysis.

**Results:**

A total of 300 outpatients with AD were enrolled. Most parents (80%) had a high instruction level. Eighty-one percent reported to have a certain amount of fear of TCS. At the multivariable analysis, fear of TCS was associated with the following items: believing that TCS treatment advantages do not overweight disadvantages (*P* = 0.011); believing that TCS may be dangerous independently from the specific side effect (*P* < 0.001). Moreover, TCS fear was associated with fear of applying too much cream (*P* = 0.001).

**Conclusion:**

TCS phobia is widespread among Italian families of children with AD. Fear of TCS is associated with fear of applying too much cream, thus increasing the risk of poor compliance and treatment failure. Therapeutic education of families on the use of TCS should be implemented.

**Electronic supplementary material:**

The online version of this article (doi:10.1186/s13052-017-0330-7) contains supplementary material, which is available to authorized users.

## Background

Atopic dermatitis (AD) is a common chronic, pruritic, inflammatory skin disease, mostly affecting children, with heterogeneous lifetime prevalence figures, ranging worldwide from 8 to 18% [1]. Although its exact pathogenesis still remains unclear, it is well established that AD depends on a combination of genetic and environmental factors. In patients with AD, a genetically determined alteration of the skin barrier allows penetration of environmental factors, which are linked to a local - and systemic - immune dysregulation [2–4]. Taking into account the inflammatory nature of the disorder and the immune-based pathogenesis of the clinical picture (pruritus, skin hyperreactivity), topical corticosteroids (TCS) have always been the cornerstone of the treatment of AD [4]. Appropriate use of TCS is characterized by a high efficacy, with reduced disease relapsing, and by a low incidence of local and systemic side effects [5, 6]. Nevertheless, it is very common, in clinical practice, to detect fear and anxiety regarding safety of TCS both among patients affected with AD and their families and among health care professionals [7, 8]. Such attitude towards TCS treatment, named TCS phobia, may lead to poor adherence to therapy and consequent treatment failure.

With the present multicenter study, we investigated prevalence and determinants of steroid phobia in a large group of Italian families of pediatric patients affected by AD.

## Methods

### Study design

This is a multicenter, cross-sectional study conducted in the dermatology units and general pediatric units of 9 pediatric centers located in 9 Italian cities (Ancona, Bologna, Cesena, Genoa, Lucca, Naples, Padova, Palermo, Rome, Bologna). The included centers represent some of the main Italian centers for pediatric dermatology.

From September 2013 to September 2014, families of pediatric patients with AD consulting for the first time at one of the above mentioned centers were consecutively enrolled. Inclusion criteria were age <18 years and a diagnosis of AD. Exclusion criteria were presence of contraindications to the use of TCS (i.e. previous sensitization to TCS, active viral illness) and presence of other forms of dermatitis.

Prior to enrolment, one caregiver was asked to sign an informed consent to the study.

The study was approved by the Bambino Gesù Children’s Hospital Ethical Committee (coordinator center) and by the Ethical Committees of all the other involved hospitals.

### Questionnaire

At enrolment, a self-filling questionnaire was given to one of the caregivers attending the patient.

The questionnaire included items on sociodemographic characteristics, previously identified atopic conditions, symptom duration, reason for the outpatient visit, clinical characteristics and SCORAD index at visit, quality of life assessment (DLQI), treatment prescriptions, previous therapeutic education.

Finally, the questionnaire included a standardized set of questions on fear of corticosteroids [7], divided in 3 sections.

In the first section, fear of TCS was investigated, based on a 10-point visual analogue scale (VAS).

In the second section, specific fears and beliefs were investigated through 21 items. The level of agreement to each item was measured through a 4-point Lickert-scale.

In the third section, frequency of parents’ behaviours regarding TCS treatment was investigated through 10 items based on a 4-point Lickert-scale.

### Clinical parameters

AD was diagnosed according to Hanifin e Rajka criteria [9]. Severity of AD was assessed through the SCORAD index [10]. Pruritus was evaluated daily for a week after enrolment using the VAS score: from 0 (for no itch) to 10 (for maximum unbearable itch).

### Statistical methods

We estimated a sample size of 302, based on an expected prevalence of TCS phobia of 80%, with a ±5% precision level and a 97% confidence interval.

The VAS scale used for expressing fear of corticosteroids was translated into categories as follows: 0–1: Not at all; 2–3: A little; 4–6: Moderately; 7–8: A lot; 9–10: Very much.

The ordinal qualitative variables (based on the 4-point Lickert scale) of the second and third section of the questionnaire were converted into quantitative, defined as a score ranging from 0 to 3 (0 representing a completely negative attitude towards TCS, 3 representing a completely positive attitude).

Additionally, we classified each item of the second and third part of the questionnaire into one category among efficacy, safety, compliance and quality of life. We then created 4 new “synthetic” variables, named after these categories. The value of the new variables was the sum of the scores of the items in each category, normalized to 100.

Proportions were calculated excluding missing values. Results of the descriptive analysis are reported as mean and SD or median and range as appropriate.

We performed a univariate analysis through simple linear regression to investigate factors associated with TCS fear, among the following: sociodemographic variables (child’s age and sex, respondent’s level of instruction); comorbidities (celiac diseases, thyroid diseases, asthma, allergic rhinitis, food allergy); clinical data including age at AD diagnosis, AD duration, AD evolution, AD treatment (emollients, systemic corticosteroids, topic TCS, systemic immunosuppressants, topic immunosuppressants, diet), SCORAD, median weekly pruritus, DQLI; having received therapeutic education; each item of the second and third section of the questionnaire and each of the synthetic variables (efficacy, safety, compliance and quality of life).

After checking for multicollinearity, multiple linear regression was used to create two multivariable models. Both models included fear of TCS as the dependent variable, and, as independent variables, those significantly associated with fear of TCS (*p* < 0.05) at the univariate analysis. The first model included only the single items of the questionnaire (that were significantly associated with the outcome at the univariate analysis). The second model included the synthetic variables. Normality was verified with Shapiro-Wilk test. Results were considered statistically significant with *p* < 0.05. Statistical analysis was performed using Stata 11.

## Results

### Patients’ characteristics

We consecutively enrolled a total of 300 patients with AD.

Sociodemographic characteristics are shown in Table 1.

**Table 1:** Patients’ demographic and clinical characteristics

Females (n, %)	143	47,7
Age (median, range)	3	0.2 - 17
Comorbidity (n, %)	83	27,7
celiac disease	4	1,3
diabetes	0	0,0
thyreopathy	2	0,7
asthma	32	10,7
rhynitis	30	10,0
food allergy	10	3,3
other	16	5,3
Years of age at AD diagnosis (median, range)	0,4	0-13
AD duration in years (median, range)	1,8	0-15
Disease evolution (n, %)		
stable	43	14,3
periodic relapses	164	54,7
worsening	68	22,7
improving	29	9,7
irregular	21	7,0
Treatment (n, %)		
moisturizers	241	84,0
topical steroids	236	82,2
systemic steroids	56	19,5
topical immunosuppressants	31	10,8
systemic immunosuppressants	10	3,5
diet	81	28,2
other	31	10,8
SCORAD (n, %)		
mild	114	39,7
moderate	125	43,6
severe	52	18,1
Therapeutic education (n, %)	201	67

Median age of patients was 3 years. 144/300 patients (48%) were females.

A large proportion of respondent caregivers had a high education level (80% had a high school or university degree).

Twenty-seven percent of the included patients had comorbidity, mainly asthma or allergic disorders (allergic rhinitis or food allergy).

Regarding AD, in most cases, the disease had been diagnosed during the first year of life (median age at AD diagnosis: 5 months), and the median AD duration at enrolment was 1.8 years. Most patients had a moderate-severe AD (61.7%). The mean pruritus score was 5 (SD: 2).

A total of 287 patients were taking a treatment for AD at enrolment. A large proportion of patients were on topical steroids (82.2%), while 84% were on moisturizers. Concerning other therapies, 56 patients were (19.5%) on systemic steroids, 31 (10.8%) on topical immunosuppressants and 10 (3.5%) on systemic immunosuppressants. Almost 30% of patients were treated with an elimination diet. Two thirds of respondents had received therapeutic education, either continuous or occasional.

**Table 2:** Response to questions on TCS specific fears and on behaviors regarding TCS use

Fears and beliefs	Category	I completely disagree	I don’t really agree	I agree to a certain extent	I completely agree
TCS are effective over a short time period	E	12 (4%)	25 (9%)	84 (30%)	155 (56%)
TCS are effective over a long time period	E	81 (30%)	58 (21%)	100 (37%)	33 (12%)
TCS pass into the bloodstream	S	66 (25%)	40 (15%)	92 (35%)	67 (25%)
TCS can lead to infections	S	147 (56%)	55 (21%)	40 (15%)	22 (8%)
TCS make you fat	S	120 (44%)	40 (15%)	69 (25%)	42 (15%)
TCS damage your skin	S	59 (22%)	44 (16%)	104 (38%)	65 (24%)
TCS will affect my future health	S	93 (35%)	51 (19%)	90 (33%)	35 (13%)
There is a dependency risk	S	90 (33%)	35 (13%)	84 (31%)	62 (23%)
I can become resistant to TCS	S	42 (16%)	44 (17%)	107 (40%)	72 (27%)
TCS become inefficient over time	E	31 (12%)	39 (14%)	110 (41%)	89 (33%)
TCS calm symptoms but don’t treat the cause	E	24 (9%)	25 (9%)	67 (24%)	159 (58%)
TCS make eczema worse	E	152 (57%)	58 (22%)	43 (16%)	15 (6%)
TCS stop the eczema from coming up to the surface of the skin	E	33 (12%)	37 (14%)	72 (27%)	129 (48%)
TCS can lead to asthma	S	181 (72%)	36 (14%)	29 (11%)	7 (3%)
I don’t know of any side-effects but I’m still afraid of TCS	S	43 (16%)	43 (16%)	102 (38%)	82 (30%)
TCS are more dangerous than CS in tablet form	S	177 (66%)	37 (14%)	35 (13%)	18 (7%)
TCS treatment takes time and effort	E	89 (32%)	74 (27%)	73 (27%)	38 (14%)
TCS treatment is complicated	Q	157 (58%)	57 (21%)	38 (14%)	19 (7%)
TCS treatment helps me improve my quality of life	Q	26 (9%)	27 (10%)	122 (44%)	100 (36%)
TCS increase my well-being	Q	36 (13%)	31 (11%)	115 (42%)	91 (33%)
The advantages of TCS use outweigh the disadvantages	Q	23 (8%)	44 (16%)	120 (44%)	87 (32%)
Behaviors regarding TCS treatment		Never	Sometimes	Often	Always
I’m afraid of applying too much cream	S	76 (29%)	114 (44%)	58 (22%)	11 (4%)
I’m afraid of using the cream for too long	S	49 (19%)	104 (40%)	77 (30%)	28 (11%)
I’m afraid of putting cream on certain zones like the eyelids, where the skin is thinner	S	47 (18%)	70 (27%)	71 (27%)	73 (28%)
It’s more dangerous to use TCS on children than on adults	S	42 (16%)	72 (28%)	75 (29%)	72 (28%)
If the doctor prescribed TCS then I would apply the prescription	C	31 (12%)	67 (26%)	53 (20%)	109 (42%)
I wait as long as I can before applying the treatment	C	72 (27%)	70 (27%)	64 (24%)	56 (21%)
I stop the treatment as soon as I can	C	34 (13%)	51 (19%)	87 (33%)	90 (34%)
I am careful to rub the cream in well when I apply it	S	11 (4%)	27 (10%)	71 (27%)	151 (58%)
I avoid putting TCS on my child’s hands	S	78 (30%)	46 (18%)	32 (12%)	102 (40%)
I need reassurance about TCS	S	44 (17%)	72 (27%)	61 (23%)	87 (33%)

*E* efficacy, *S* safety, *Q* quality of life, *C* compliance

Mean DLQI score was 3.

### Descriptive and multivariate analysis

Overall, a high proportion of patients reported having fears regarding TCS treatment: at the VAS scale, “No fear at all” was reported by 18.8% of the enrolled patients only (A little: 26.4%, Moderately: 36.8%; A lot: 13.7%; Very much: 4.3%). Responses to the other questionnaire items are reported in (Table 2).

The multivariable analysis identified few items significantly associated with fear of TCS treatment: believing that TCS treatment advantages do not overweigh disadvantages (*P* = 0.011); believing that TCS may be dangerous independently from the specific side effect (*P* < 0.001); moreover, TCS fear was associated with fear of applying too much cream (*P* = 0.001). Results of univariate and multivariate analyses are reported in Additional file 1.

When subgrouping the items into categories, items concerning safety were more associated with fear compared with items concerning efficacy, compliance or quality of life.

## Discussion

In the present study, we show that, in Italy, fear of TCS is very common among families of pediatric patients affected with AD.

We investigated corticophobia in an exclusively pediatric population. Only few previous studies on TCS phobia have been conducted on a population of children [11–14].

According to our results, fear of TCS is not motivated by specific reasons: most respondents reported that, according to them, TCS are dangerous independently from the specific side effects, and the treatment disadvantages are not balanced by its advantages. In previous studies, the predominant concerns about TCS were cutaneous side-effects and failure to thrive [7, 15, 16].

Old generation TCS had a high systemic absorption rate, especially in children [17, 18]. Therefore, their long-term or inadequate use commonly caused a variety of local and systemic side effects, including folliculitis, erythema, reduced skin thickness and/or skin atrophy, striae rubrae, Cushing-like syndrome [19]. Such side effects are still stigmatized in the perception of the patients regarding this type of drug, despite the excellent safety profile of new generation TCS [19, 20]. Moreover, when appropriately used, new generation TCS are highly effective: they reduce inflammation, itching and relapse rate, thus dramatically improving the quality of life of the patients and their families.

Our study also shows that fear of TCS is related to the fear of applying too much cream. Thus, corticophobia may trigger a vicious circle leading to treatment failure: a reduction of compliance may cause prolonged and frequent relapses, which reduce trust in the treatment, thus further decreasing compliance.

Several studies highlighted the important role of the patient-doctor relationship in order to reduce fear of TCS [7, 11, 14–16]. We only investigated the effect of need of reassurance from the patient’s doctor on TCS fear, and did not find any association. Nevertheless, as, according to our results, corticophobia is specifically directed towards treatment safety, it is mandatory that family pediatricians and general physicians dedicate a consistent time of the visit to identifying the patients’ fear of TCS, in order to address misconceptions, thus improving the families’ trust and compliance through evidence-based information.

Interestingly, 82% of our population was under TCS treatment, demonstrating that, despite fear, the parents were compliant to the prescription of their caregivers. Nevertheless, it is not possible to prove whether the use of TCS was adequate.

A large proportion of participants (two thirds) had received a therapeutic education for the management of atopic dermatitis. However, therapeutic education did not seem to affect fear of TCS among participants. In Italy, a therapeutic patient education program has been introduced in the last 5–10 years, in the third-level centers participating in the study. This education program aims at informing the families of pediatric patients about the disease course and at empowering them towards an autonomous therapeutic management of the disease [21]. The therapeutic education includes information on the appropriate modalities for applying topical products and on the use of distraction techniques aimed at creating a relaxing environment and at transforming the dressing moment into a pleasant interaction between the child and his parent [22–24]; no specific information is actually given on the safety profile of TCS. This may explain the lack of association between therapeutic education and TCS fear. Therapeutic education sessions could actually be excellent contexts for addressing TCS fear with participating families, thus improving their knowledge on safety and effectiveness of new generation TCS, with a subsequent, possible reduction of TCS fear.

The present study has several limitations. The main limitation concerns selection bias. The patients were enrolled from amongst those presenting to the pediatric dermatology outpatient clinics of the above mentioned hospitals. Nevertheless, this is a multicenter study involving hospitals that are located in different Italian regions, therefore the sample should be sufficiently representative of the Italian population of pediatric patients with AD. Moreover, only a very limited number of families refused to answer to the questionnaire, nevertheless we cannot exclude that patients whose parents refused to answer to the questionnaire differed in terms of age and sex from those that agreed to enrolment. Another limitation concerns the score used to assess TCS phobia. We adopted the score proposed by Aubert-Wastiaux [7]. The same score subsequently informed the production of a newer scale [25], which had good psychometric properties, but was not available at the time of our study. The adoption of such scale might have yielded more precise results.

## Conclusion

AD is a chronic disorder and its treatment, mainly based on topical agents, is a long-term “therapeutic management”, rather than a common therapy. Awareness of AD treatments’ side effects is crucial, nevertheless, truths and misconceptions regarding this issue should be precisely addressed in order to ensure compliance. A specific educational program should follow a holistic approach and establish a strong relationship between the patient’s family and caregivers. In fact, the results of our study prove the need of implementing and focusing the therapeutic education on the use of TCS and to spread it to all professionals involved in the management of AD. It is mandatory that caregivers dedicate time to inform parents about the safety of the new generation products, whose main advantage is a clearly improved risk/benefit ratio.

## Additional file

Additional file 1: Univariate and multivariate analysis results (dependent variable: TCS fear). (DOCX 22 kb)

## Abbreviations

AD: Atopic dermatitis
DLQI: Dermatology life quality index
SCORAD: Scoring atopic dermatitis
TCS: Topical corticosteroids
VAS: Visual analog scale
