# Supplementary material for: Topical corticosteroid phobia in parents of pediatric patients with atopic dermatitis: a multicentre survey
Source: Ital J Pediatr. 2017 Feb 28;43:22. doi: 10.1186/s13052-017-0330-7 (PMC5330138; doi:10.1186/s13052-017-0330-7)
Supplement: Additional file 1: — Univariate and multivariate analysis results (dependent variable: TCS fear). (DOCX 22 kb) [file 13052_2017_330_MOESM1_ESM.docx]

Supplementary material: univariate and multivariate analysis results (dependent variable: TCS fear).

|  | **Univariate analysis** | | | **Multivariate analysis** | | | |
| --- | --- | --- | --- | --- | --- | --- | --- |
|  | **coeff** | **95% CI** | **p** | **coeff** | **95% CI** | **p** |  |
| Age (years) | -0.05 | -0.13 to 0.02 | 0.139 | - | - | - |  |
| Sex | 0.01 | -0.63 to 0.66 | 0.968 | - | - | - |  |
| High school diploma | 0.27 | -0.48 to 1.04 | 0.474 | - | - | - |  |
| Comorbidity | -0.63 | -1.28 to 0.03 | 0.062 | - | - | - |  |
| celiac disease | -2.59 | -5.07 to -0.13 | **0.039** | - | - | - |  |
| thyreopathy | -2.58 | -6.07 to 0.91 | 0.147 | - | - | - |  |
| asthma | -0.76 | -1.7 to 0.17 | 0.112 | - | - | - |  |
| rhynitis | -0.38 | -1.41 to 0.66 | 0.476 | - | - | - |  |
| foodallergy | -0.19 | -1.96 to 1.58 | 0.831 | - | - | - |  |
| other | 0.07 | -1.2 to 1.34 | 0.917 | - | - | - |  |
| Years of age at AD diagnosis (median, range) | -0.18 | -0.34 to -0.02 | **0.030** | - | - | - |  |
| AD duration in years (median, range) | -0.03 | -0.11 to 0.06 | 0.534 | - | - | - |  |
| Disease evolution |  |  |  |  |  |  |  |
| stable | -0.28 | -1.14 to 0.58 | 0.517 | - | - | - |  |
| periodicrelapses | 0.04 | -0.57 to 0.64 | 0.905 | - | - | - |  |
| worsening | 0.39 | -0.32 to 1.1 | 0.277 | - | - | - |  |
| improving | -0.55 | -1.59 to 0.49 | 0.295 | - | - | - |  |
| irregular | -0.69 | -1.81 to 0.44 | 0.230 | - | - | - |  |
| Treatment |  |  |  |  |  |  |  |
| moisturizers | -0.8 | -1.54 to -0.07 | **0.032** | - | - | - |  |
| topical steroids | -0.55 | -1.28 to 0.17 | 0.134 | - | - | - |  |
| systemic steroids | -0.24 | -1 to 0.52 | 0.532 | - | - | - |  |
| topical immunosuppressants | 0.25 | -1.34 to 1.84 | 0.759 | - | - | - |  |
| systemic immunosuppressants | 0.12 | -0.84 to 1.09 | 0.801 | - | - | - |  |
| diet | 0.16 | -0.51 to 0.83 | 0.643 | - | - | - |  |
| SCORAD | 0.001 | -0.01 to 0.02 | 0.929 | - | - | - |  |
| Median weekly pruritus | -0.02 | -0.19 to 0.15 | 0.815 | - | - | - |  |
| DQLI | -0.43 | -0.93 to 0.07 | 0.093 | - | - | - |  |
| Therapeutic education | 0.023 | -0.72 to 0.77 | 0.951 | - | - | - |  |
|  |  |  |  |  |  |  |  |
| TCS are effective over a short time period | -0.12 | -0.48 - 0.23 | 0.491 | - | - | - |  |
| TCS are effective over a long time period | -0.32 | -0.61 to -0.39 | **0.026** | - | - | - |  |
| TCS pass into the bloodstream | 0.41 | -0.68 to -0.15 | **0.003** | - | - | - |  |
| TCS can lead to infections | -0.01 | -0.31 – 0.3 | 0.962 | - | - | - |  |
| TCS make you fat | -0.51 | -0.76 to -0.25 | **<0.001** | - | - | - |  |
| TCS damage your skin | -0.54 | -0.81 to -0.26 | **<0.001** | - | - | - |  |
| TCS will affect my future health | -0.81 | -0.1 to -0.54 | **<0.001** | - | - | - |  |
| There is a dependency risk | -0.32 | -0.57 to 0.07 | **0.013** | - | - | - |  |
| I can become resistant to TCS | -0.27 | -0.57 to 0.24 | 0.072 | - | - | - |  |
| TCS become inefficient over time | -0.32 | -0.62 to -0.01 | **0.047** | - | - | - |  |
| TCS calm symptoms but don’t treat the cause | -0.36 | -0.66 to -0.54 | **0.021** | - | - | - |  |
| TCS make eczema worse | -0.35 | -0.67 to -0.03 | **0.031** | - | - | - |  |
| TCS stop the eczema from coming up to the surface of the skin | -0.3 | -0.58 to -0.01 | **0.040** | - | - | - |  |
| TCS can lead to asthma | 0.38 | -0.77 to 0.01 | 0.053 | - | - | - |  |
| I don’t know of any side-effects but I’m still afraid of TCS | -1.01 | -1.27 to -0.74 | **<0.001** | -0.76 | -1.05 to -0.46 | **<0.001** |  |
| TCS are more dangerous than CS in tablet form | -2.05 | -0.52 to 0.11 | 0.203 | - | - | - |  |
| TCS treatment takes time and effort | -0.11 | -0.4 to 0.17 | 0.421 | - | - | - |  |
| TCS treatment is complicated | -0.33 | -0.64 to -0.02 | **0.040** | - | - | - |  |
| TCS treatment helps me improve my quality of life | -0.28 | -0.47 to -0.09 | **0.003** | - | - | - |  |
| TCS increase my well-being | -0.45 | -0.75 to -0.15 | **0.003** | - | - | - |  |
| The advantages of TCS use outweigh the disadvantages | -0.69 | -1.01 to -0.37 | **<0.001** | -0.46 | -0.8 to -0.1 | **0.011** |  |
| I’m afraid of applying too much cream | -1.01 | -1.35 to -0.67 | **<0.001** | -0.66 | -1.05 to -0.28 | **0.001** |  |
| I’m afraid of using the cream for too long | -0.87 | -1.19 to -0.56 | **<0.001** | - | - | - |  |
| I’m afraid of putting cream on certain zones like the eyelids, where the skin is thinner | -0.4 | -0.68 to -0.13 | **0.004** | - | - | - |  |
| It’s more dangerous to use TCS on children than on adults | -0.63 | -0.9 to -0.36 | **<0.001** | - | - | - |  |
| If the doctor prescribed TCS then I would apply the prescription | 0.04 | -0.23 to 0.32 | 0.742 | - | - | - |  |
| I wait as long as I can before applying the treatment | -0.52 | -0.79 to -0.26 | **<0.001** | - | - | - |  |
| I stop the treatment as soon as I can | -0.39 | -0.68 to -0.11 | **0.008** | - | - | - |  |
| I am careful to rub the cream in well when I apply it | -0.26 | -0.61 to -0.1 | 0.160 | - | - | - |  |
| I avoid putting TCS on my child’s hands | -0.1 | -0.34 to 0.13 | 0.373 | - | - | - |  |
| I need reassurance about TCS | -0.81 | -1.07 to -0.56 | **<0.001** | - | - | - |  |
|  |  |  |  |  |  |  |  |
| Efficacy | -0.32 | -0.48 to -0.02 | **<0.001** | - | - | - |  |
| Safety | -0.04 | -0.06 to -0.03 | **<0.001** | - | - | - |  |
| Quality of life | -0.03 | -0.09 to -0.03 | **<0.001** | - | - | - |  |
| Compliance | -0.02 | -0.03 to -0.01 | **0.003** | - | - | - |  |
